# Wolf Creek XVIII Part 4: innovations in ECPR technology^☆^

**DOI:** 10.1016/j.resplu.2026.101274

**Published:** 2026-02-19

**Authors:** Georg Trummer, Demetris Yannopoulos, Yohei Okada

**Affiliations:** a Department of Emergency Medicine, University Medical Center Freiburg, Faculty of Medicine, University of Freiburg, Freiburg, Germany; b Center for Resuscitation Medicine, MN Mobile Resuscitation Consortium, University of Minnesota Medical School, United States; c Prehospital and Emergency Research Centre, Health Services and Systems Research Duke-NUS Medical School, National University of Singapore, Singapore

## Abstract

The 50th Anniversary Wolf Creek XVIII Conference was hosted by the Max Harry Weil Institute for Critical Care Research and Innovation in Ann Arbor, Michigan, USA on June 19–21, 2025. “Innovations in ECPR Technology” was one topic of focused presentation and discussion by invited panelists and conference participants made up of international academic and industry scientists as well as thought leaders in the field of cardiac arrest resuscitation.

This panel was part of the conference agenda in order to update the auditorium on the current state of the art of ECPR increasingly offered by specialized centers worldwide but still far away from routine use. In view of the ongoing high mortality and morbidity of patients treated with CPR following cardiac arrest, ECPR arises as a potential promise to improve this challenge, however many questions with respect to patient selection, implementation and the required related technical and educational resources are currently not solved and remain as relevant barriers. Moreover, current ECPR does not follow standardized protocols and is therefore a highly individualized therapy of each performing center. This is a relevant barrier in order to conduct trials with larger and more homogenous groups of patients. Despite the tempting option to overcome the shortcomings of CPR, the field of ECPR primarily requires targeted research with focus on community studies and the rationale implementation of extracorporeal circulation in the CPR scenario.

## Introduction

Sudden cardiac arrest is one of the leading causes of death in industrialized countries, with an average incidence of 84 per 100,000 inhabitants per year.^1^ Data from US, Japan and Germany indicates a cardiac origin in 60–69% of out-of-hospital cardiac arrests (OHCA).^2^, ^3^, ^4^ Despite prompt initiation of cardiopulmonary resuscitation (CPR), the vast majority of patients do not survive cardiac arrest with frequent neurologic sequela in the few survivors. Within the chain of survival and the scenario of prolonged CPR, return of spontaneous circulation (ROSC) is the major milestone. However, if ROSC fails to occur, treatment options towards successful resuscitation are very limited due to the ongoing circulatory failure.

In this situation, implementing extracorporeal cardiopulmonary resuscitation (ECPR) as ultimate chance towards survival has become a potential option in the recent decade.^5^, ^6^ However, despite being a potentially life-saving therapeutic approach, one has to recognize that ECPR adds complexity to the scenario of prolonged CPR, demanding additional resources and a specific level of expertise across all involved professional groups. In view of the increasing dynamic development of ECPR in different countries and regions, the outline for the panel on”Innovations in ECPR Technology” was set and staffed with three panelists from Europe, Asia and America contributing a current update and future visions on the role of ECPR in resuscitation, as discussed at the 50th Anniversary Wolf Creek XVIII Conference. This review aims to summarize the discussions held at this conference, particularly the panel on “Innovations in ECPR Technology,” and to highlight strategies and challenges for the future implementation and establishment of ECPR.

## Methods

Since its inception in 1975, the Wolf Creek Conference has a well-established tradition of providing a unique forum for robust intellectual exchange between thought leaders and scientists from academia and industry focused on advancing the science and practice of cardiac arrest resuscitation. The 50th Anniversary Wolf Creek XVIII Conference was hosted by the Max Harry Weil Institute for Critical Care Research and Innovation in Ann Arbor, Michigan, USA on June 19–21, 2025. Meeting invitees included international academic and industry scientists as well as thought leaders in the field of cardiac arrest resuscitation. All participants were required to complete conflict of interest disclosures. “Innovations in ECPR Technology” was one of six focused panel topics that was presented and discussed during the conference. The presentations by invited panelist and discussion among all meeting participants focused on the current state, potential future state, knowledge gaps, barriers to translation, and research priorities. As indicated above, the three panelists represented the state of the art of ECPR with their specific research and clinical background:

Dimitri Yannopoulos, Center for Resuscitation Medicine at the University of Minnesota Medical School and Principal Investigator of the ARREST-trial, the first randomized study on ECPR published in 2020.

Yohei Okada, a researcher at Duke-NUS Medical School in Singapore, and an emergency physician/intensive care physician and former research fellow at Kyoto University in Japan, contributing his specific experience from a region of the world, where ECPR has already been performed in the late 2000s.

Georg Trummer, cardiac surgeon at the Department of Emergency Medicine University Hospital Freiburg/Germany. The research of his group set the baseline for the outline of the CARL therapy (Controlled Automated Reperfusion of the whoLe body), which is a comprehensive and physiology guided approach in the field of ECPR.

The three panelists at the session on ECPR gave a detailed presentation on the status of the use of extracorporeal circulation in patients with refractory outcomes. In addition, speakers from America, Europe, and Asia reported on specific approaches and their views on the possible further development and integration of ECPR in the field of cardiopulmonary resuscitation.

## Current state

ECPR is a rescue therapy that has been gradually introduced in selected centers over the past 20 years for use in cases of unsuccessful resuscitation.^7^, ^8^, ^9^, ^10^ The central aspect of this therapeutic approach is the replacement of circulatory function in the absence of ROSC with compact extracorporeal circulation, namely as Extracorporeal Membrane Oxygenation (ECMO). The implementation of this therapy follows the beginnings of ECMO therapy in the 1970s, when elements of heart–lung machines (tube system, oxygenator, blood pump, drive unit) were combined into a single unit, primarily in the field of cardiac surgery, in order to bridge circulatory failure for hours or days in cases of post-cardiotomy failure.^11^, ^12^ The increasing availability of compact and portable ECMO systems from the 2000s onwards led to an expansion of their use beyond cardiac surgery and, consequently, to their use in CPR in patients who were no longer able to achieve spontaneous circulation and were therefore in immediate danger of losing their lives.^13^, ^14^

Early adopters of this therapy were initially hospitals in Taiwan, Japan, and Korea, followed by centers in Europe and the United States. While early publications on ECPR were largely limited to reports of case series, in recent years there has been an increasing trend toward describing the scope of application and potential benefits of ECPR in randomized controlled trials. For example, in the year 2020, the Paris working group led by Burgouin described a large patient series with *N* = 525 patients who had received ECPR (389 cases in-hospital setting, 136, prehospital setting) and compared them with 12,666 patients who had undergone conventional resuscitation during the same period.^15^ No survival advantage was observed for patients who received ECPR, which is most likely related to the liberal patient selection. Regardless of this, this work impressively describes the feasibility of ECPR in the out-of-hospital setting, which was subsequently implemented in other centers. Another milestone was the publication of the AREEST trial in 2020 by Yannopoulos' working group in Minnesota, which was the first positive randomized controlled trial to investigate the benefits of ECPR compared to conventional CPR. In contrast to the study from Paris, this controlled study involved a much more rigorous patient selection process. After *N* = 30 patients had already been enrolled, the study was terminated due to the clear superiority of ECPR versus CPR in terms of survival (ECPR 43% vs. CPR 7%).^16^

Another key study in the field of ECPR is the Prague OHCA study, which was published in 2022. This work is remarkable in several respects, as it describes not only the use of ECMO in resuscitation cases, but also a whole package of measures that was initially implemented in emergency medical services and hospital care in the greater Prague area.^17^ After including *N* = 256 patients over a period of 7 years, the study was also terminated by the safety board due to futility. Neurological intact survival was demonstrated in 31.5% of patients in the ECPR group, however there was no statistically significant difference compared to patients receiving conventional resuscitation, of whom 22% survived. Although the study was underpowered, it should be noted that some patients from the standard therapy group were treated with ECPR as a life-saving therapy in the sense of a crossover, which ultimately had a direct influence on the statistically significant study results.

Another prospective, randomized study evaluating ECPR is the INCEPTION trial which was conducted in the Netherlands and published in the New England Journal of Medicine in 2023.^18^ This multicenter study included 160 patients with out-of-hospital cardiac arrest who were randomized to receive conventional resuscitation or CPR in the hospital. The results showed no advantage for treatment with ECPR, with a survival rate of 20% in both the conventional and intervention groups. The reasons for this are the subject of much debate. Among other things, there was a delay in starting cannulation and connection to extracorporeal circulation compared to the studies described above, reflecting relevant accompanying factors such as logistics and the experience of the teams involved.

## Specific contribution of the panelists within the panel “Innovations in ECPR Technology”

Demetris Yannopoulos focused in his presentation on neurologically intact survival, which is possible if circulation can be restored within 45 min. The central aspect here is overcoming continued circulatory failure through extracorporeal circulation, in which conventional resuscitation methods prove ineffective after a resuscitation period of more than 30 min, even in cases of shockable heart rhythms. This strategy is supported by a meta-analysis published in Lancet in 2023 involving over 9000 patients, which showed that ECPR reduces hospital mortality and improves neurologically intact survival compared to conventional resuscitation.^19^

This is confirmed in a joint data analysis of the ARREST and Prague OHCA studies, which describes a clear survival advantage with ECPR for patients with a shockable heart rhythm, regardless of the time of implantation.^20^ Regardless of this, however, it is obvious that cannulation of patients with OHCA after transport to the hospital generally prevents early cannulation, so that the majority of patients can only be connected to extracorporeal circulation after 45 min.^21^ Against this background, the “TIME” trial in Minneapolis is about to start, which is planned for the end of 2025. This study aims to generate further evidence for the correct choice of timing for ECPR implementation in patients undergoing continued resuscitation. Closely linked to the challenge of performing extracorporeal circulation implantation in a timely manner is the question of whether the connection to the ECMO should take place inside or outside the hospital. In conclusion, Yannopoulos presents the concept of out-of-hospital ECMO implantation developed by the Minnesota Mobile Resuscitation Consortium (MMRC), in which specialized teams perform implantation with compact ECMO systems out-of-hospital, directly at the site of the emergency, in suitable patients, significantly reducing the low-flow time to enable more neurologically intact survival of the affected patients.

The subsequent presentation was given by Georg Trummer from the University Medical Center Freiburg. He began by explaining that extracorporeal circulation was historically used as a rescue procedure in the 1970s for patients facing imminent death, particularly in cases of post-cardiotomy failure. Building on this, since the 2000s, this therapeutic concept has also become established outside of surgical units and, since the 2010s, has also been referred to as ECPR in cases of acute cardiac arrest. Although this method is being prepared for use in specialized clinics worldwide, ECPR is still not a standardized therapy but rather a combination of CPR-ALS and VA ECMO that is still very much controlled by individual centers and users. This refers to the concept of targeted reperfusion after cardiac arrest, which Georg Trummer's working group has been studying experimentally and clinically for the past 20 years.^22^, ^23^, ^24^ The concept is based on the understanding that cardiac arrest followed by resuscitation measures physiologically corresponds to severe and generalized ischemia reperfusion injury of the entire body. Based on extensive animal and clinical data, the Freiburg working group developed the CARL-Therapy, which aims to minimize reperfusion damage through immediate monitoring and controlled reperfusion solution and reperfusion conditions. This application is based on extracorporeal circulation that was specifically developed to meet the clinical requirements described above. For example, part of the CARL concept involves closely monitoring and controlling oxygen during the reperfusion phase, measuring blood pressure from the start of extracorporeal circulation, and ensuring perfusion of the brain with high and pulsatile blood flow. This concept was first established in a case series and a multicenter study and showed neurologically intact survival in 27.5% of patients despite a very long median low-flow time of 59 min before connection to the CARL system.^25^ Currently, the therapeutic approach is extended in another experimental setting towards exsanguinating circulatory arrest. In summary, CARL is a rational based ECPR concept targeting various physiological targets in the scenario of CPR followed by extracorporeal circulation.

The third presenter (Yohei Okada, Duke-NUS Medical School, Singapore) presented on the current ECPR practice in Japan. The Japanese experience with ECPR dates back to the late 1980s, and by the mid-2010, approximately 2–3% of all OHCA cases each year were provided with ECPR.^9^ He then described the situation in Osaka prefecture based on findings from Osaka-CRITICAL study, where approximately 130–160 patients are treated with ECPR annually.^10^ Similar to the situation in Europe and the USA, the major challenge is to initiate ECPR as early as possible within the first 60 min after cardiac arrest. The general time course in Osaka showed a median time from emergency call to ECPR of around 53 min (interquartile range 45–64 min).^26^ He also shared several approaches to shorten the time to ECPR initiation in Japan. One potential factor contributing to the relatively widespread use of ECPR is that in about half of the facilities, cannulation is performed by emergency physicians, not only by cardiologists.^27^ In some cases, patients are brought directly into the angiography suite, while in others, cannulation is performed in the emergency department using ultrasound guidance. Furthermore, the introduction of hybrid emergency departments equipped with fluoroscopy-enabled resuscitation bays has been reported to further shorten the time to ECPR initiation.

In prehospital settings, the revised protocol in Tokyo, Japan for refractory ventricular fibrillation was presented. In this protocol, the number defibrillation attempts are limited up to three, with the aim of prioritizing conveyance to a suitable center with ECMO capacity. In addition, it was also reported that physician-staffed ambulances and helicopters enable earlier patient contact by emergency physicians and timely decision-making, thereby allowing preparation for ECPR initiation. Furthermore, in Utsunomiya City, a prehospital ECPR program has been introduced, representing another approach to shorten the time to ECPR initiation.^28^, ^29^

## Potential future state

**Fig. 1:**
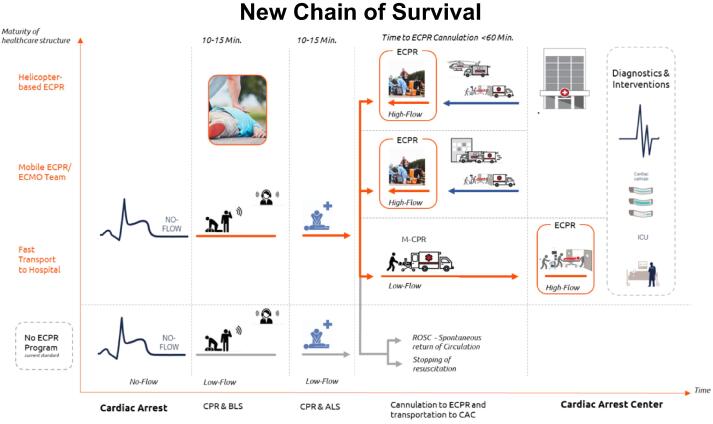
**New Chain of Survival**. Goal of this “New Chain of Survival” is the minimization of No-flow times before potential connection to ECPR. Current state of the art is CPR at scene with transport to a suitable hospital, where options for ECPR are may available. The next level accelerates transport to the hospital (load-and-go strategy) to reach a center of ECPR as soon as possible. More advanced systems carry specialized ECPR teams to the scene, either ground or helicopter based providing ECPR therapy at the initial scene of CPR.

Concluding the global overview of this session, the rise of ECPR reflects the urge and vision of the medical community to improve the continued insufficient outcome of patients suffering from acute cardiac arrest. Core of this approach is the overcome of the ongoing circulatory failure, which is frequently inherent to a cardiac cause of the circulatory standstill, therefore primarily opening a window for further diagnostic and goal directed therapeutic intervention. This also relates to a modified chain of survival where CPR-ALS has been augmented with the option of ECPR in or out of the hospital as new subsequent chain link (Fig. 1). Dependent on country- and regional-specific structures, adding such an element requires a well-balanced system of implementation, since many involved stakeholders along the chain of survival have to be considered. Exemplary for this challenge is the timely information of ECPR teams and specialized hospitals, when Emergency Services are dispatched to OHCA patients. Despite a substantial number of OHCA patients does not meet the indication for ECPR, therefore initially rather not of interest of ECPR teams and hospitals, an early (pre-)alert allows these providers a more targeted and tailored allocation of their resources.

Beyond that, resources are a keyword in the field of ECPR. The implementation of ECPR into the scenario of CPR bears the risk of waste, if applied according to CPR as “method and tool for everybody”. Although upcoming element of a new chain of survival, patient selection will differ for various reasons from the preceeding, CPR inherent all-embracing approach towards a best and comparable strict selection of patients.

Furthermore, from the physiologic point of view, the implementation of extracorporeal circulation into the scenario of cardiac arrest offers options towards a sophisticated control of blood circulation. Especially against the background of a generalized ischemia/reperfusion injury of the brain, heart and the body, such an approach could potentially limit the frequent sequela of CPR related post resuscitation syndrome and the related morbidity and mortality.

## Knowledge gaps and priorities for research and implementation

– Integration and timing of ECPR into the CPR-ALS scenario
– Implementation of ECPR in or out of the hospital in OHCA patients
– Considerations regarding establishment of a simple, basic perfusion (“extracorporeal support”) versus an individualized, targeted perfusion in terms of an “extracorporeal therapy”
– Scientific evaluation and publication of the aforementioned objectives in the specific surrounding of research in CPR with its inherent heterogeneity

Up to date, ECPR has been offered as ultimate alternative in selected centers worldwide only, corresponding conclusive evidence is rare. Moreover, integration of ECPR into the algorithm of CPR-ALS and the run of extracorporeal circulation are still very individual, user specific and not standardized. Together with the aforementioned objectives, these create a multiparametric field of subsequent research. The given complexity for research and the adjacent scientific accompanied implementation of this new therapy in this field is high. Such challenges may relate to ethical considerations how study designs and informed consent may be appropriate in the moment of an acute life-threatening situation or even require training and education of hospital and EM teams up front.

## Conclusions

The continued unsatisfying outcome of patients with CPR motivated emergency and intensive care teams around the world to implement ECPR into the therapeutic approach of these patients. Although the corresponding guidelines mentioned such an approach already in 2010, many questions and processes around CPR/ECPR remain unsolved and assign ECPR still as rare therapeutic offer in specialized centers in the US, some European, Asian and Australian hospitals. However, the comparable short ECPR history comprising around 20 years evolves stepwise structures and proceedings that could set the baseline for the future. One example for this approach is the “TIME” study from the Minneapolis group, who will investigate the correct choice of timing for ECPR implementation in patients undergoing continued resuscitation. Closely linked to the challenge of performing extracorporeal circulation implantation in a timely manner is the question of whether the connection to the ECMO should take place inside or outside the hospital. The Japanese experience, as early adopters already active over decades in this field, showed impressively how ECPR programs may be established as part of CPR routine. The aspects of a physiologic guided, targeted and therefore therapeutic reperfusion (CARL) within ECPR is another approach which has been created on extensive physiologic research and is on its way to be continued and evaluated in further laboratory and clinical research using.

In summary, ECPR remains a controversy but also fascinating field of critical care. Refined engineering of the extracorporeal circulatory systems in combination with accordingly designed research and studies will elucidate and path the way for the future of ECPR.

## CRediT authorship contribution statement

**Georg Trummer:** Writing – review & editing, Writing – original draft, Visualization, Validation, Project administration, Formal analysis, Data curation, Conceptualization. **Demetris Yannopoulos:** Writing – review & editing, Visualization, Validation, Conceptualization. **Yohei Okada:** Writing – review & editing, Writing – original draft, Visualization, Validation, Supervision, Project administration, Methodology, Conceptualization.

## Declaration of competing interest

Georg Trummer is consultant for Resuscitec GmbH.

Demetris Yannopoulos states no COI.

Yohei Okada had received a research grant from the Zoll foundation.
